# Glyphosate internal dose estimation: comparing passive dosimetry and biomonitoring in simulated heavy residential herbicide application

**DOI:** 10.2478/aiht-2026-77-4101

**Published:** 2026-06-30

**Authors:** Daniel G. Kougias, Rongcan Sun, Kenneth M. Unice, Eric W. Miller, Jennifer Pierce, Michael Kovochich

**Affiliations:** K2 Toxicology and Integrated Risk Assessment, Las Vegas, NV, USA; Syneos Health Consulting, Morrisville, NC, USA; TRC Companies, Pittsburgh, PA, USA; Benchmark Risk Group, Chicago, IL, USA

**Keywords:** dermal exposure, inhalation exposure, pesticides, risk assessment, urinalysis, analiza mokraće, dermalna izloženost, pesticidi, procjena izloženosti, procjena rizika

## Abstract

Glyphosate exposure is commonly assessed using either passive dosimetry or biomonitoring. This study tests the hypothesis that internal dose estimates derived from passive dosimetry are higher and more uncertain than those derived from biomonitoring by comparing concurrently collected passive dosimetry and biomonitoring data from a controlled simulation of heavy residential herbicide application. The two predominant exposure routes were evaluated separately: six applicators were protected from dermal exposures with hooded Tyvek^®^ coveralls and gloves and six were protected from inhalation exposure with a respirator. Urine was serially collected from all applicators between 30 min prior to and up to 36 h after application, while breathing-zone air samplers (left and right lapel) and four dermal patches were collected from dermally protected applicators. Internal doses were independently estimated from air and dermal patch measurements (passive dosimetry) and from urinary glyphosate residues (biomonitoring; dilution-adjusted). Average total internal daily doses of glyphosate estimated from passive dosimetry were approximately seven times greater than biomonitoring-based estimates. This overestimation appeared attributable to the dermal pathway and was consistent with the uncertainty in the assumed dermal absorption and clothing penetration factors as well as with the uncertainty introduced by extrapolating a limited number of dermal patch measurements to larger body regions. Even so, the highest individual internal daily dose of glyphosate derived from passive dosimetry remained below the internal-dose benchmark derived from conservative regulatory health-based guidance values (60 µg/kg/day), suggesting a low likelihood of adverse health effects under the tested conditions. By directly comparing concurrently collected passive dosimetry and biomonitoring data in applicators with detectable urinary glyphosate, this study provides empirical support for using biomonitoring to evaluate and refine dermal passive-dosimetry assumptions in glyphosate exposure assessment.

## Abstract (hr)

Izloženost glifosatu obično se procjenjuje primjenom pasivne dozimetrije ili biomonitoringa. U ovom je istraživanju testirana hipoteza da su procjene interne doze izvedene iz pasivne dozimetrije više i nesigurnije od onih dobivenih biomonitoringom, i to usporedbom istodobno prikupljenih podataka pasivne dozimetrije i biomonitoringa iz kontrolirane simulacije intenzivne primjene herbicida u kućanstvu. Pritom su zasebno procijenjena dva prevladavajuća puta izloženosti: šest primjenitelja otopine glifosata bilo je zaštićeno od dermalne izloženosti kombinezonima Tyvek^®^ s kapuljačom i rukavicama, a šest je nosilo kratke hlače i majicu, ali i zaštitnu masku s respiratorom radi zaštite od izloženosti glifosatu udisanjem. Mokraća je prikupljana od svakog primjenitelja, počevši od 30 minuta prije primjene pa do 36 sati nakon nje. Usto je svaki primjenitelj u zaštitnom kombinezonu nosio četiri vanjska dermalna jastučića i dva uzorkivača zraka (lijevi i desni) u zoni disanja. Interne doze neovisno su procijenjene na temelju mjerenja iz zraka i s dermalnih jastučića (pasivna dozimetrija) te iz ostataka glifosata u mokraći (biomonitoring). Prosječne ukupne interne dnevne doze glifosata procijenjene pasivnom dozimetrijom bile su približno sedam puta veće od procjena temeljenih na biomonitoringu. Ova se procijenjenost može pripisati dermalnom putu unosa te je u skladu s nesigurnošću u pretpostavljenim čimbenicima dermalne apsorpcije i prodiranja kroz odjeću, kao i s nesigurnošću uvedenom ekstrapolacijom ograničenog broja mjerenja s dermalnih jastučića na veće dijelove tijela. Unatoč tomu, najviša pojedinačna interna dnevna doza glifosata izvedena iz pasivne dozimetrije ostala je ispod referentne vrijednosti interne doze izvedene iz konzervativnih regulatornih zdravstvenih smjernica (60 µg/kg/dan), što upućuje na nisku vjerojatnost štetnih učinaka na zdravlje u testiranim uvjetima. Izravnom usporedbom pasivne dozimetrije i biomonitoringa ovo istraživanje empirijski podupire odabir biomonitoringa za evaluaciju i ugađanje pretpostavki za bolju procjenu izloženosti glifosatu na temelju pasivne dozimetrije.

Glyphosate was commercially introduced as an herbicidal active ingredient in 1974. Since the advent of genetically modified glyphosate-tolerant crops in the late 1990s, glyphosate has become the most widely used herbicide in the US and worldwide (1, 2). For those who spray glyphosate-containing herbicides, dermal absorption, despite being low (i.e., <1 % fractional dermal absorption), has been identified as the primary exposure route (3, 4), whereas inhalation and incidental ingestion only account for a small portion of the total exposure (5).

Public and regulatory interest in glyphosate exposure has grown due to its widespread use and conflicting health risk assessments. In 2015, the International Agency for Research on Cancer (IARC) classified glyphosate as probably carcinogenic to humans (6), a classification that spurred substantial scientific and regulatory debate. In contrast, multiple national and international regulatory and public-health agencies, including the US Environmental Protection Agency, the Agency for Toxic Substances and Disease Registry, Health Canada, the European Food Safety Authority, the European Chemicals Agency, the Joint FAO/WHO Meeting on Pesticide Residues, the Australian Pesticides and Veterinary Medicines Authority, and the Food Safety Commission of Japan, have concluded that glyphosate is not likely to pose a carcinogenic risk to humans at typical exposure levels (3, 4, 7,8,9,10,11,12). These divergent evaluations underscore the importance of accurately characterising human glyphosate exposure under realistic use scenarios, particularly for high-end residential applications, to inform exposure assessment and risk characterisation.

Exposure can be measured by either passive dosimetry or biological monitoring (biomonitoring) techniques. The former quantifies external deposition of the chemical and the latter determines concentrations of the parent compound and/or its metabolites in bodily fluids (13). Both can be used to estimate the internal (systemic) dose of a chemical after exposure under different assumptions, but biomonitoring is recognised as the gold standard, since it most accurately captures the cumulative dose integrated across routes of exposure by measuring an actual, as opposed to a potential, amount of an absorbed chemical (14, 15). The known toxicokinetics of glyphosate support the use of biomonitoring for exposure assessment of glyphosate, as the absorbed glyphosate does not undergo extensive metabolism in humans [i.e. less than 1 % is metabolised into aminomethylphosphonic acid (AMPA)], does not bioaccumulate, and is predominantly and rapidly excreted in urine (7). Such toxicokinetic characteristics support the use of urinary biomonitoring as an exposure assessment tool for acute, short-term exposures. Passive dosimetry, in turn, relies on a number of assumptions (e.g. clothing penetration, fractional absorption, body surface areas of different anatomical regions, and uniform deposition) and is valuable in characterising dermal deposition patterns, clothing protection factors, and route-specific contributions to internal dose, all of which may be generalised to other chemicals and facilitate the development of targeted interventions to reduce exposure. Passive dosimetry is also easier to conduct, less intrusive, and more cost-effective than biomonitoring (13). Therefore, although biomonitoring is generally acknowledged to be more accurate in assessing systemic dose, the use of both biomonitoring and passive dosimetry to assess exposure may elucidate key differences in each methodology and assumptions in estimating internal doses.

Earlier comparisons between the two methods in occupational settings found that exposure-derived dose estimates were of similar magnitude to biomonitoring-derived doses, but the two methods did not rank job classes in the same order from highest to lowest estimated exposure (16). Such findings have been interpreted in light of validation studies showing that biological monitoring can more directly reflect systemic dose under conditions where absorption and elimination are well characterised, because it integrates actual absorbed dose across exposure routes rather than potential external deposition (13, 15). However, such combined assessments using both passive dosimetry and biomonitoring remain inadequate for glyphosate, despite its well-characterised absorption and elimination. To date, there is only one published study that has used both passive dosimetry and biomonitoring on the same study population to estimate internal doses of glyphosate (17). Although its results showed that passive dosimetry generally overestimated the internal dose compared to biomonitoring in conifer seedling nursery workers, the authors used dated study-specific assumptions that overestimate both clothing penetration and dermal absorption, only assessed dermal exposure for passive dosimetry, and, most importantly, did not detect glyphosate in any of the urine samples and, therefore, estimated internal doses from urinary glyphosate concentrations postulated to be 0.005 µg/mL (i.e. one-half the lower limit of method validation).

In view of these shortcomings, this study was designed to address a key methodological gap by directly comparing internal dose estimates derived from passive dosimetry and biomonitoring obtained from the population studied by Pierce et al. (18) and by Kougias et al. (19). The first study reported glyphosate air sampling and dermal patch data, whereas the second derived internal dose estimates from urinary glyphosate and AMPA concentrations in the same study population. Using the air sampling and dermal patch data from the first study, the present study estimates internal glyphosate doses with applicator-specific anthropometric measures and current evidence-based assumptions for clothing penetration and dermal absorption.

By evaluating whether traditional patch- and air-based exposure measurements systematically overestimate systemic dose relative to urinary biomonitoring, this study aimed to clarify the strengths, limitations, and underlying assumptions of each approach. The overarching objective was to improve the interpretation and application of exposure assessment methods for glyphosate and other pesticides with similar toxicokinetic profiles, particularly in research and regulatory contexts where accurate estimation of internal dose is critical.

## PARTICIPANTS AND METHODS

### Study protocol

**Figure 1:**
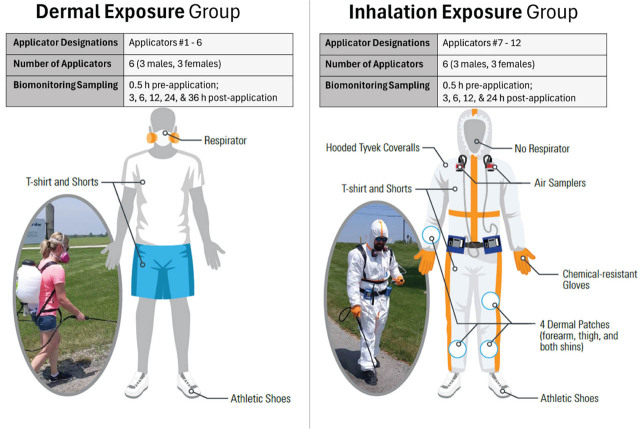
Study design schematic for dermal versus inhalation exposure groups. Left: Dermal exposure group, in which applicators wore typical summer clothing (t-shirt, shorts, and athletic shoes) and a half-face respirator, with no dermal patches applied because the skin was directly exposed. Right: Inhalation exposure group, in which applicators wore Tyvek^®^ coveralls covering the arms, legs, and torso, along with chemical-resistant gloves, but no respirator. Four dermal patches were placed on the outside of the coveralls at the shins, thigh, and forearm (shown as blue-outlined circles) to measure potential dermal deposition. All other aspects of the exposure scenario, including spray equipment, product concentration, application duration, and total volume applied, were identical for both groups

The evaluation of inhalation and dermal exposure to glyphosate reported previously (18) was approved by the Institutional Review Board of Advarra, Inc., Columbia, MD, USA (protocol No. Pro00036892). All participants provided written informed consent prior to participation. In brief, the study involved 12 adult participants (“applicators”) of both sexes, all employees of Cardno ChemRisk (Chicago, IL, USA) (now Stantec), divided into two exposure groups with three men and three women in each. Apart from sex, the groups did not match in age or body mass index. There was no crossover between dermal- and inhalation-focused exposure scenarios – one group was protected from inhalation exposure to evaluate dermal exposure, whereas the other was protected from dermal exposure to evaluate inhalation exposure – because a crossover design would have required sufficient wash-out periods to avoid carryover effects. The dermal exposure group wore shorts, t-shirts, athletic shoes, and 3M 60921 half-face respirators with OV/AG/P100 cartridges (3M, St. Paul, MN, USA), while the inhalation exposure group wore identical clothing under hooded Tyvek^®^ coveralls (DuPont de Nemours, Wilmington, DE, USA) as well as chemical-resistant gloves but no respirator (Figure 1).

The study was conducted in Monee, IL, USA on a single day in July of 2019, during which temperatures were 25.2–31.9 °C and relative humidity was 40–62 %. Per manufacturer’s specifications, each applicator mixed about 300 mL of Roundup^®^ Weed & Grass Killer Super Concentrate (Bayer CropScience LP, St. Louis, MO, USA; EPA Reg. No. 71995-25) containing glyphosate with about 15 L of water and loaded into a Roundup^®^ Commercial backpack sprayer (Bayer CropScience LP), resulting in a 0.96 % glyphosate solution. The solution was then continuously sprayed along the ~200-m perimeter of a gravel and asphalt yard until empty. Two applicators from each exposure group were spraying at a time. Each applicator repeated this process four times, which corresponded to a total exposure time of 100 min (3–5 min for mixing/loading and 20–22 min for spraying times four). Following the 100-min exposure, applicators washed their hands with soap and water per instructions on the product’s container.

Total exposure duration of 100 min was selected to conform to the minimum air sampling duration specified by the OSHA Method PV2067 and was not intended to represent typical residential consumer use durations, which are expected to be shorter than in this study. Therefore, each applicator sprayed a total of ~60.57 L of diluted product over 100 min, which represents a heavy-use application scenario. To mitigate variability across participants, exposure conditions were tightly controlled, including the use of a uniform gravel/asphalt perimeter to provide a consistent application surface, identical spray equipment and application protocols, and real-time oversight by trained helpers assigned to each applicator to ensure that the four concurrent applicators sprayed at approximately the same rate, maintained even spacing while walking the perimeter, and sprayed for the same duration, thereby minimising additional spray drift exposure.

### Biomonitoring

#### Urine sampling and analysis

All 12 applicators provided urine samples 30 min prior to mixing/loading/spraying (pre-application) and 3, 6, 12, and 24 h after finishing spraying (post-application). The six applicators in the dermal exposure group also provided 36-h post-application urinary samples due to the possible delay in absorption. The urine samples were analysed for glyphosate and AMPA by the Health Research Institute (Fairfield, IA, USA) using high-performance liquid chromatography-triple quadrupole mass spectrometry (HPLC-MS/MS) and ISO 17025:2005 standard-accredited testing methods. For glyphosate in urine, the limit of detection (LOD) and limit of quantification (LOQ) were 0.02 ng/mL and 0.05 ng/mL, respectively. As for AMPA, the LOD and LOQ were 0.013 ng/mL and 0.05 ng/mL, respectively. Levels were adjusted for dilution effects using urine specific gravity.

As detailed in Kougias et al. (19), total urine output across the 38.167-h study period was estimated rather than measured. Applicators recorded their dietary intake, including all liquids, starting with the morning of application and continuing through the final bladder emptying. Fluid and food intake were logged for each interval between urine collections to identify potential anomalies in urinary glyphosate concentrations and to provide context relevant to urinary output. For internal dose estimation, a body-weight-adjusted urine production rate of 1.5 mL/kg/hour was applied to estimate urine volume for each collection interval. Across applicators, the resulting total urinary output approximated 2–4 L/day, which is a relatively high daily urine output, consistent with the documented fluid intake and physical activity during the study.

#### Internal dose calculations

The urinary glyphosate and AMPA concentrations reported by Pierce et al. (18) were used to calculate internal doses as reported in Kougias et al. (19). In short, we used the urinary concentrations of total glyphosate residues (“effective glyphosate”) to calculate internal doses and to account for any potential losses of glyphosate due to metabolism. Specifically, the urinary “effective glyphosate” concentrations from the 3, 6, 12, and 24 h post-application spot urine samples were used because these consecutive samples most closely approximated a daily urinary output period, corresponding to 26 h and 10 min of estimated urinary output, and captured the primary window of elevated urinary glyphosate residues. The 36-h samples were not included in the internal dose calculation because they fell outside this defined period, and concentrations at 36 h had returned to levels that were not statistically different from pre-application concentrations.

For a given spot urine sample, the “effective glyphosate” concentration was multiplied by the corresponding assumed urinary output volume, divided by a toxicokinetic recovery value of 0.82 approximated from data in Figure 2 of Wester et al. (20) and then by self-reported body weight. This 0.82 recovery factor reflects the assumption that 82 % of the absorbed glyphosate would be recovered in urine within the monitoring period. For each applicator, the total internal daily dose was obtained by summing the internal doses for each selected post-application spot urine sample measurement. We made no adjustment for detectable concentrations in pre-application samples.

**Figure 2:**
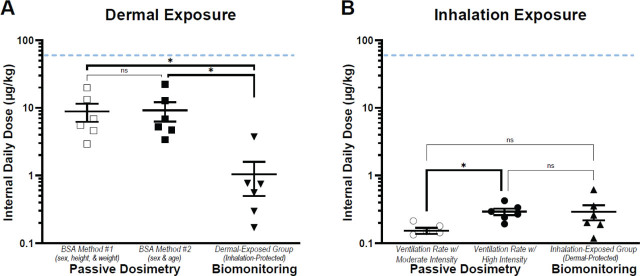
Different techniques and assumptions in estimating internal daily doses of glyphosate originating from A) dermal exposure and B) inhalation exposure. Bars represent mean ± SEM. The blue-dotted line represents the internal dose benchmark derived from the most conservative regulatory health-based guidance value established for glyphosate. *p<0.05; ns: not statistically significant

Since total urinary output volume was not measured directly but high daily fluid intakes were recorded across all the applicators, a precautionary body weight-specific urine production rate of 1.5 mL/kg/h was used to calculate individual urine output volumes across 26 h and 10 min. The estimated total urinary output over this period was apportioned across the intervals represented by the 3-, 6-, 12-, and 24-h post-application spot urine samples in proportion to the elapsed time between sample collections. This interval-specific urinary volume was then multiplied by the corresponding urinary “effective glyphosate” concentration to estimate the amount of glyphosate excreted during each interval. Because internal dose was calculated as the product of measured urinary concentration and the assumed urine volume (normalised to body weight), using a higher assumed urine output increases estimated internal dose, rendering this approach conservatively biased toward overestimation of the internal dose. The associated uncertainty is addressed further in the Discussion.

#### Baseline (background) dose calculation

Using the same assumptions, we estimated the baseline internal dose for each applicator based on the “effective glyphosate” concentration from the urine sample collected 30 min prior to spraying (pre-application). This dose represented background exposure.

### Passive dosimetry

#### Dermal patch sampling and analysis

As described previously (18), immediately before the start of the study, one applicator wearing hooded Tyvek^®^ coveralls used the backpack sprayer to apply water containing a blue spray pattern indicator (Liquid Harvest Lazer^TM^, Sanco Industries Inc., Fort Wayne, IN, USA). Colour markings on the coveralls indicated highest depositions on the shins (i.e., the anterior portion of the leg distal to the knee), the dorsal side of the forearm ipsilateral to the hand operating the spray wand, and the proximal portion of the anterior thigh contralateral to the hand operating the spray wand. Accordingly, one circular (142 mm in diameter) borosilicate glass fibre patch was placed on each of these four locations for all applicators in the inhalation exposure group during the 100-min exposure scenario to assess dermal deposition of glyphosate and “worst-case” exposure.

Patch samples were analysed for glyphosate by Bureau Veritas (Novi, MI, USA), a laboratory accredited by the American Industrial Hygiene Association (AIHA), using HPLC with an ultraviolet detector (HPLC-UV) according to a modified OSHA Method PV2067 (21). For each sample, the mass-based reporting limit (RL) was 1.0 µg, which corresponded to an area density-based RL of approximately 0.0063 µg/cm^2^.

#### Air sampling and analysis

For each of the six applicators in the inhalation exposure group, two breathing zone air samples (i.e. from the left and right lapel) were collected at a flow rate of 1.0 L/min (±6 %) for 100 min (±2 min) of exposure. All 12 air samples were collected on glass fibre filters and were analysed as described above for patch samples. For each sample, the mass-based RL was 0.1 µg, which corresponds to a concentration-based RL of approximately 0.001 mg/m^3^.

### Internal dose calculation

The dermal and inhalation contributions to the internal dose of glyphosate derived from passive dosimetry (i.e. dermal patch and air sampling data, respectively) were summed for each individual applicator to estimate total internal daily dose.

#### Dermal component of internal dose

The estimated internal dose resulting from dermal exposure for each individual applicator was calculated based on the dermal patch data extrapolated to applicator-specific regional body surface areas and adjusted for clothing and dermal penetration using the following equation:

[1] $$\mathrm{Internal}\;{\mathrm{dose}}_{\mathrm{dermal}}=\sum \left(C_{\mathrm{patch}}\times {\mathrm{BSA}}_{\mathrm{regional}}\times \mathrm{CPF}\times f_{\mathrm{dermal}}÷\mathrm{BW}\right)$$

where Internal dose_dermal_ is the internal dose resulting from dermal exposure (µg/kg/day), C_patch_ is the glyphosate patch concentration (µg/cm^2^), BSA_regional_ is the regional body surface area (cm^2^), CPF is the clothing penetration factor, f_dermal_ is dermal bioavailability or absorption factor, and BW is the applicator’s body weight (kg).

The total body surface area (BSA) was partitioned into eight body regions, namely the head, trunk, upper arms, forearms, hands, thighs, lower legs, and feet, based not only on dermal patch location but also on clothing protection considerations and available BSA data from the 2011 Exposure Factors Handbook (22) (Table 1). BSAs were estimated using two different methods. Method #1 used applicator-specific sex and anthropometric measures (height and weight) to estimate adult-based regional BSAs, while Method #2 used applicator-specific sex and age group to estimate regional BSA based on means from a larger dataset. For both methods, it was assumed that 45 % of the arm’s surface area was attributed to the forearms and 55 % to the upper arms. Additionally, for Method #2, which provided a regional BSA for the legs (including both the thighs and lower legs), it was assumed that 40 % of the leg’s surface area was attributed to the lower legs and 60 % to the thighs.

**Table 1:** Apportioned body regions and the corresponding patch, clothing protection, and assumed clothing penetration factor used in estimations of internal dose resulting from dermal exposure for each individual applicator

**Apportioned Body Region**	**BSA Method #1 (sex, height, weight)**	**BSA Method #2 (age & sex)**	**Patch Used**	**Clothing Protection**	**Clothing Penetration Factor**	**Dermal Absorption Factor**
Head	✓	✓	minimum of all four patches	No	1	0.01
Trunk	✓	✓	minimum of all four patches	Yes	0.1049	0.01
Upper arms	†	†	minimum of all four patches	Yes	0.1049	0.01
Forearms	†	†	forearm	No	1	0.01
Hands	✓	✓	forearm	No	1	0.01
Thighs	✓	†	thigh	Yes	0.1049	0.01
Lower legs	✓	†	average of lower leg patches	No	1	0.01
Feet	✓	✓	average of lower leg patches	Yes	0.1049	0.01

† Using data from the 2011 Exposure Factors Handbook (22), a regional BSA for both arms entirely (i.e., not separately for the upper arms and forearms) was provided; therefore, to adequately apply a clothing protection factor for the upper arms and exclude it from the forearms as the applicators wore t-shirts, it was assumed that 45 % of the arm’s surface area was attributed to the forearms and 55 % to the upper arms (22). Similarly, for the dataset used in Method #2, which only provided a regional BSA for both legs entirely and not separately for the thighs and lower legs, it was assumed that 40 % of the leg’s surface area was attributed to the lower legs and 60 % to the thighs (22)

Glyphosate concentrations found on the four dermal patches were selectively extrapolated to these specific areas of the body based on proximity and conservative assumptions. Specifically, glyphosate concentrations from the patch on the dorsal side of the forearm of the spraying arm was extrapolated to the forearms and hands, the patches on the shins to the lower legs and feet, and the patch on the thigh opposite the spraying arm to the thighs. Because no dermal patches were placed on the head, trunk, or upper arms, the minimum glyphosate concentration measured across the four dermal patches was used as a surrogate for these regions to avoid extrapolating higher localised deposition measurements to unpatched body areas expected to have lower direct deposition.

Clothing protection was assumed for the trunk, upper arms, thighs, and feet in the regionally apportioned calculations of the internal dose, as this is representative of the clothing worn by the dermal exposure group applicators and was intended to represent limited clothing during warm-weather application. Calculations pertaining to these clothing-protected areas included a clothing penetration factor (CPF) of 0.1049 based on a mean fractional clothing penetration of 10.49 % reported for single-layer clothing in applicators described in the US EPA’s Pesticide Handlers Exposure Database (23), while no adjustment (i.e., a CPF of 1) was applied for bare skin areas (Table 1).

A dermal absorption factor of 0.01, corresponding to 1 % fractional dermal absorption, was applied in all regionally apportioned internal dose calculations, consistent with conservative dermal absorption assumptions used in regulatory glyphosate risk assessments (3, 4). Furthermore, for each applicator, all regionally apportioned calculations of the internal dose were divided by the applicator’s self-reported body weight.

#### Inhalation component of internal dose

The estimated internal dose resulting from inhalation exposure for each individual applicator was calculated based on the air sampling data and sex-, age group-, and activity level-specific, body weight-adjusted ventilation rates from descriptive statistics specified in the 2011 Exposure Factors Handbook (24) using the following equation:

[2] $$\mathrm{Internal}\;{\mathrm{Dose}}_{\mathrm{inhalation}}=C_{\mathrm{air}}\times R_{p}\times 100\times f_{\mathrm{inhalation}}$$

where Internal Dose_inhalation_ is the internal dose resulting from inhalation exposure (µg/kg/day), C_air_ is the glyphosate air sample concentration averaged from the left and right lapel (µg/m^3^), R_p_ is the average ventilation rate adjusted for body weight [m^3^/(min·kg)], 100 is exposure duration in minutes, and f_inhalation_ is the inhalation bioavailability or absorption factor.

The 50^th^ percentile body weight-adjusted average ventilation rate was selected for each participant based on sex, age group, and activity category (24). The applicable age groups were 21 to <31 and 31 to <41. Although the applicators herein walked at a relatively slow pace of approximately 0.3 m/s, the combined physical demands of carrying the backpack sprayer, operating the hand pump, and continuously applying product with the spray wand supported classification of the activity as moderate to high intensity, consistent with adult physical activity intensity classifications from the American College of Sports Medicine and the American Heart Association (see 25). Therefore, for each applicator, inhalation-derived internal dose was calculated under two activity-intensity scenarios, using the 50^th^ percentile body weight-adjusted average ventilation rates for moderate- and high-intensity activity, respectively. For inhalation absorption, a factor of 1 was applied, assuming complete absorption through the respiratory pathways as a precautionary worst-case assumption (26).

### Comparison of biomonitoring- and passive dosimetry-derived internal dose estimates

Biomonitoring- and passive dosimetry-derived internal dose estimates were compared in a route-specific manner. Internal doses estimated from dermal patch data were compared with biomonitoring-derived internal doses from the dermal exposure group, while internal doses estimated from air sampling data were compared with biomonitoring-derived internal doses from the inhalation exposure group. These route-specific comparisons were used because dermal and inhalation exposures were evaluated separately in two groups of applicators with distinct personal protective equipment.

For summary-level comparison with route-combined passive dosimetry estimates and regulatory health-based guidance values, an approximate route-combined biomonitoring-derived internal dose was calculated by summing the average biomonitoring-derived internal dose from the inhalation exposure group with that from the dermal exposure group. Since inhalation exposure and dermal exposure were assessed separately in two different groups and because biomonitoring estimates aggregate absorbed exposure, this sum of averages across the two groups double counts background contributions and may also include any incidental exposures that may have occurred (e.g. dermal exposures to the uncovered face or through the athletic shoes in the inhalation exposure group or inhalation exposures after the application period in the dermal exposure group). Therefore, this route-combined biomonitoring-derived internal dose was considered a conservative estimate for comparison to regulatory health-based guidance values and passive dosimetry estimates.

### Risk assessment

For risk assessment, a total internal daily dose of glyphosate derived from passive dosimetry was estimated for each applicator by summing internal doses estimated from dermal patch and air sampling results. These total internal doses were compared against the most conservative regulatory health-based guidance value: the acceptable daily intake (ADI) value of 0.3 mg/kg/day established by both the Australian Pesticides and Veterinary Medicines Authority (APVMA) and the Pest Management Regulatory Agency (PMRA) of Health Canada (8, 11).

To compare internal doses from simulated heavy application with this external oral guidance value, the ADI was adjusted using a correction factor of 20 % to account for limited oral absorption, yielding an internal dose comparator of 0.06 mg/kg/day, or 60 µg/kg/day. This absorption assumption has been used in prior glyphosate risk assessments (27) and endorsed by the European Food Safety Authority (EFSA) (4) as a conservative estimate within the range of oral absorption values (7–36 %) reported by the Agency for Toxic Substances and Disease Registry (ATSDR) (7).

### Statistical analysis

All analyses were run on GraphPad Prism version 10.4.0 for Windows (GraphPad Software, Boston, MA, USA). Given the route-specific comparison groups, differences between internal dose estimates were evaluated using the Kruskal-Wallis H test followed by Dunn’s multiple comparison test. All data are presented as means ± standard errors of the mean (SEM).

## RESULTS

Aside from the previously reported summary statistics for the passive dosimetry data (18), here we report applicator-specific dermal patch and air sampling results along with estimated BSAs and ventilation rates (Table 2).

**Table 2:** Applicator-specific information from the six applicators in the inhalation exposure group, including estimated total body surface areas (BSAs), body weight-adjusted ventilation rates (*R*_*p*_), and glyphosate concentrations determined in dermal patch and breathing zone air samples

	**Applicator #7**	**Applicator #8**	**Applicator #9**	**Applicator #10**	**Applicator #11**	**Applicator #12**	
Sex	Male	Female	Male	Female	Male	Female	
*Dermal considerations*							
Age group	30s	30s	30s	20s	20s	20s	
Estimated BSA (cm^2^): Method #1	23,335	17,510	24,040	15,454	21,868	15,310	
Estimated BSA (cm^2^): Method #2	22,449	18,149	22,449	17,756	21,915	17,756	
*Inhalation considerations*							
Age group	31 to <41	31 to <41	21 to <31	21 to <31	21 to <31	21 to <31	
R_p_ (m^3^/min-kg) for moderate-intensity activities	0.000344	0.000304	0.000345	0.000316	0.000345	0.000316	
R_p_ (m^3^/min-kg) for high-intensity activities	0.000625	0.00059	0.000644	0.000627	0.000644	0.000627	
*Sample location*	*Dermal patch glyphosate concentrations (µg/cm^2^)*						*Average*
Left shin^a^	37.89	2.72	5.75	6.95	2.40	36.62	*15.39*
Right shin^a^	42.94	6.19	28.41	5.62	0.95	2.46	*14.43*
Forearm^b^	0.055	7.58	0.19	0.76	16.42	25.89	*8.48*
Thigh^c^	0.82	2.53	0.57	0.63	22.10	35.99	*10.44*
*Average*	*20.43*	*4.75*	*8.73*	*3.49*	*10.47*	*25.24*	*12.18*
*Sample location*	*Breathing zone air glyphosate concentrations (µg/m^3^)*						*Average*
Left lapel	4.4	3.5	5.2	5.7	4.8	7.5	*5.2*
Right lapel	3.3	3.0	3.1	3.7	5.6	6.0	*4.1*
*Average*	*3.9*	*3.3*	*4.2*	*4.7*	*5.2*	*6.8*	*4.7*

a The anterior portion of the leg distal to the knee;

b The dorsal side of the forearm of the spraying arm;

c The anterior thigh opposite the spraying arm

The average glyphosate surface loadings measured on each region-specific dermal patch across all applicators were similar in magnitude (8.48–15.39 µg/cm^2^), and no consistent patterns of regional deposition were found in the four patches across all applicators. Glyphosate concentrations in breathing-zone air samples ranged from 3.0 to 7.5 µg/m^3^, with no consistent left-right differences between lapel samples. Visual inspection of the data showed no apparent relationship between breathing-zone air concentrations and dermal patch glyphosate levels across applicators.

### Dermal internal dose estimates

The Kruskal-Wallis H test revealed a statistically significant difference among the three dermal internal daily glyphosate dose estimates: biomonitoring-derived doses and passive dosimetry-derived doses calculated using the two BSA methods [H(2)=10.14, *p*=0.0022]. Dunn’s multiple comparison test indicated that this difference was attributable to higher passive dosimetry-derived estimates relative to biomonitoring-derived estimates, not to the method used to calculate BSA. Specifically, passive dosimetry-derived estimates were not sensitive to BSA calculation method (Method #1: 8.8±2.6 µg/kg/day; Method #2: 9.2±2.9 µg/kg/day; p>0.9), and both were roughly nine times greater than the biomonitoring-derived estimate for the dermal exposure group (1.0±0.5 µg/kg/day; *p*<0.02) (Figure 2A).

### Inhalation internal dose estimates

The Kruskal-Wallis H test also revealed a statistically significant difference among the inhalation-related internal daily glyphosate dose estimates [H(2)=7.45, *p*=0.0172], but Dunn’s multiple comparison test indicated that this difference was between the passive dosimetry estimates calculated using high- versus moderate-intensity ventilation rates (0.29±0.03 µg/kg/day vs. 0.15±0.02 µg/kg/day, respectively; *p*<0.03). Irrespective of the assumed ventilation rate, passive dosimetry-derived estimates did not significantly differ from biomonitoring-derived estimates in the inhalation exposure group (0.29±0.07 µg/kg/day; *p*>0.1) (Figure 2B).

Interestingly, Applicator #10 was the only applicator in the inhalation exposure group with a substantially higher biomonitoring-derived internal daily dose than the corresponding air-sampling-derived passive dosimetry estimate. Because biomonitoring reflects aggregate absorbed exposure rather than inhalation exposure alone, incidental dermal exposure in this applicator cannot be ruled out, including potential exposure to the uncovered face, through athletic shoes, or from an inconspicuous breach in the gloves or Tyvek^®^ coveralls. This observation did not alter the group-level comparison between passive dosimetry- and biomonitoring-derived estimates for the inhalation exposure group.

### Total internal doses

**Figure 3:**
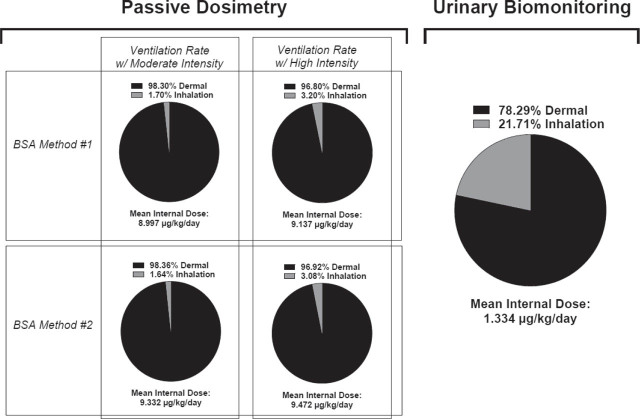
Mean total internal doses of glyphosate estimated from summing those derived from dermal patch data and air sampling data across the different methods and ventilation rates (passive dosimetry) alongside an approximate route-combined biomonitoring-derived internal dose estimated by summing mean internal doses from the dermal and inhalation exposure groups

Figure 3 shows the mean route-combined internal doses of glyphosate estimated from passive dosimetry by summing dermal patch-derived and air sampling-derived internal doses across the different BSA methods and ventilation-rate assumptions, as well as the approximate route-combined biomonitoring-derived internal dose calculated by summing the mean biomonitoring-derived internal doses from the dermal and inhalation exposure groups. In a worst-case scenario simulating heavy residential application, passive dosimetry-derived internal dose estimates were roughly seven times greater than those derived from biomonitoring. Even so, the highest individual total internal dose of glyphosate derived from passive dosimetry (22.591 µg/kg/day) was still well below the internal-dose benchmark of 60 µg/kg/day derived from the most conservative regulatory health-based guidance values (8, 11), suggesting that adverse health effects are unlikely under the tested high-end residential application scenario.

Both passive dosimetry and biomonitoring indicated that most of the internal dose was attributable to dermal exposure rather than inhalation. Passive dosimetry estimated a 1.6–3.2 % contribution from inhalation and a 96.8–98.4 % contribution from dermal exposure. Biomonitoring, in turn, estimated a 21.7 % contribution from inhalation and a 78.3 % contribution from dermal exposure.

Based on urinary biomonitoring across all 12 applicators, the average baseline internal daily dose of glyphosate estimated from pre-application urine samples was approximately 0.074 µg/kg/day, or 11 % of the average internal dose from the exposure scenario. Mean background levels accounted for approximately 7 % of the mean internal daily dose in the dermal exposure group and approximately 25 % in the inhalation group. However, mean background internal doses were similar between the dermal and inhalation exposure groups (0.076 and 0.071 µg/kg/day, respectively), suggesting that differences between groups in internal daily dose were attributable to exposure during the study rather than differences in baseline levels.

## DISCUSSION

To our knowledge, this is the first study to compare internal glyphosate doses estimated from both biomonitoring and passive dosimetry data within the same study population that 1) has detectable urinary glyphosate residue concentrations and/or 2) considers inhalation exposures. Our findings suggest that studies or reviews relying only on passive dosimetry for dose reconstruction should consider potential overestimation of the dermal dose.

In practice, passive dosimetry approaches for dermal exposure assessment, including patch-based sampling with extrapolation to whole-body surface area, are widely used in occupational hygiene and pesticide exposure assessments because they are logistically feasible in field settings and can be implemented when biomonitoring data are unavailable or impractical to collect.

For glyphosate specifically, numerous occupational studies have relied on dermal and/or inhalation-based passive dosimetry to characterise applicator exposure across forestry, agricultural, and amenity use scenarios, including studies of backpack, tractor-mounted, and controlled-droplet application systems (e.g. 5, 17, 28, 29–35). Such exposure estimates are routinely applied by industry and regulatory agencies to characterise handler and applicator exposure, evaluate the effectiveness of personal protective equipment and work practices, and inform risk-management decisions, including acceptable use patterns, label-required controls, and margin-of-exposure determinations. Consequently, understanding the extent to which internal dose estimates derived from passive dosimetry align with those derived from biomonitoring is important for interpreting exposure assessments that directly inform regulatory policy and risk-management decisions.

Even with the use of refined assumptions for dermal absorption (i.e., 1.0 % fractional dermal absorption) and clothing penetration (i.e., a CPF of 0.1049) in our study, passive dosimetry overestimated the internal dose by roughly seven times compared to biomonitoring. This overestimation resulted exclusively from the dermal patch-derived estimates, whereas air sampling-derived internal dose estimates were comparable to or even lower than biomonitoring-derived estimates in the inhalation exposure group. With respect to dermal exposure, the source of the approximate nine-fold difference between passive dosimetry- and biomonitoring-derived internal dose estimates is not precisely known. This overestimation may reflect the use of only four dermal patches placed on areas of highest anticipated dermal deposition, combined with extrapolation of those patch measurements to larger body regions under the assumption of uniform regional deposition. Although patch locations were selected based on visual assessment under the same equipment and setting, regional deposition patterns across the four patches were inconsistent across applicators. Despite identical exposure conditions and spraying instructions, these irregularities suggest that subtle behavioural factors, such as spills, pouring or mixing technique, and nozzle distance from the applicator or ground, may have influenced deposition patterns. As a result, the selected patch locations may not have consistently represented the areas of highest dermal deposition for all applicators.

According to Chester (36), depending on whether dermal patches capture *non-uniform, random deposition*, passive dosimetry can either significantly under- or overestimate exposure. Because passive dosimetry did not underestimate dermal exposure relative to biomonitoring in any applicator, sources of overestimation beyond patch placement may also have contributed. It is worth noting, however, that some assumptions used in the regionally apportioned dermal dose calculations would be expected to reduce, rather than inflate, passive dosimetry-derived estimates. For example, the lowest glyphosate concentration measured across the four dermal patches was used as the surrogate value for the head, trunk, and upper arms (Table 1), which is generally consistent with dermal exposure patterns observed among backpack/knapsack spray applicators (29, 32, 33, 37). In addition, a clothing protection factor was applied to the entire upper arms and thighs, although, as shown in Figure 1, the shorts and t-shirts worn by applicators generally did not fully cover these areas. As for other assumptions that could account for the overestimation of the internal dose resulting from dermal patch data, it is unlikely that the applicator-specific estimations of BSA played a role. The two methods for estimating BSA yielded trivial changes to internal dose estimates. Overestimation may also have been attributable to the assumption that dermal absorption and/or clothing penetration were higher than actual values. Using similar assumptions to those used in the present study, Solomon (38, 39) compared glyphosate exposure estimates from biomonitoring and passive dosimetry data collected from the literature and unpublished reports provided by the Monsanto Company and found a similar bias toward overestimation by passive dosimetry. Solomon concluded, “It is unlikely that this bias results from consistent errors in measuring the area of the dosimeters or the mass and body area of the applicator; it is more likely that the value of 1 % penetration of glyphosate through skin and/or the default value of 10 % penetration through clothing is incorrect.” Apart from US EPA’s support of <1 % human dermal penetration for glyphosate and EFSA’s “conservative” use of 1 % in risk assessment (3, 4), recent evidence supports a fractional dermal absorption of less than 1 % (40).

### Study limitations

In addition to the assumptions discussed above, this study has several limitations that should be considered when interpreting the findings.

First, this was a controlled exposure study with 12 participants allocated to a single exposure pathway (dermal or inhalation) using a parallel-group rather than crossover design, although a crossover design could have increased statistical power and controlled for inter-individual variability. Furthermore, participants were matched by sex but not by age or body mass index. In addition, because participants were employees of an organisation that conducts exposure and risk assessments, they may have been more attentive to chemical handling and protocol adherence than typical residential applicators. These factors may limit generalisability, but the controlled exposure conditions and standardised application protocol support the internal validity of the comparative assessment.

Second, although the exposure scenario was intended to represent a realistic high-end residential application, certain aspects may not reflect typical residential use. The gravel/asphalt perimeter was selected to provide a uniform and consistent application surface for all applicators and to minimise variability in spray conditions, but this setting does not capture potential interactions with vegetation, such as contact with treated foliage or surface retention on grass, which could influence dermal exposure. Conversely, the study design involved four applicators operating concurrently along a defined perimeter, which may have resulted in some degree of spray drift exposure between applicators that would not typically occur during an individual residential application. Because applicators moved at an approximate pace of 0.3 m/s around a 200-m perimeter, overlap with previously treated surfaces could occur within a few minutes of continuous application, potentially resulting in additional contact with retained residues. In addition, the exposure duration and total volume applied were selected to meet air-sampling requirements and to represent a high-end residential use scenario. Each applicator continuously applied a total of 60.6 L of diluted product over 100 min, which exceeds typical residential consumer use and, in many instances, occupational use, as reviewed in (19). Therefore, although the gravel/asphalt setting may have underestimated vegetation-related dermal contact, the application duration, total volume applied, and potential inter-applicator spray drift likely made the overall exposure scenario conservative relative to typical residential applications.

Third, the biomonitoring-based dose estimation involved uncertainties. Urinary glyphosate concentrations were adjusted for dilution using urine specific gravity, which accounts for hydration-related variability in spot samples and is an accepted alternative to creatinine normalisation. However, total urine volume output was not measured directly; instead, it was estimated using an assumed body weight-adjusted urine production rate based on documented fluid intake and activity level. Because internal dose was calculated as the product of measured urinary concentration and assumed urine volume, use of a relatively high assumed output may have conservatively overestimated internal dose if true urine output was lower. While this uncertainty affects the precision of dose estimates, it does not alter the conclusion regarding differences between passive dosimetry- and biomonitoring-derived doses and, if anything, reinforces this conclusion, given the potential for conservative bias in the biomonitoring-based estimates.

Finally, the patch-level heterogeneity discussed above highlights the limitation of relying on a small number of dermal patches to extrapolate whole-body exposure, as estimates may be sensitive to whether patches intercept areas of higher or lower deposition. Such variability likely contributes to the overestimation of internal dose observed for passive dosimetry and underscores the importance of cautious interpretation of absolute dose values derived from patch-based methods. Taken together, these limitations warrant caution in extrapolating absolute dose estimates beyond the study conditions.

## CONCLUSION

Our study showed that, under a scenario simulating heavy residential application, passive dosimetry-derived internal dose estimates were approximately seven times higher than biomonitoring-derived estimates. Even so, the highest individual passive dosimetry-derived internal dose of glyphosate (22.6 µg/kg/day) remained below the internal-dose benchmark of 60 µg/kg/day derived from the most conservative regulatory health-based guidance value, suggesting low concern for adverse health effects under the tested conditions. Although passive dosimetry has limitations for estimating glyphosate internal dose, it may serve as a precautionary method for estimating worst-case glyphosate exposure scenarios.

Still, further research is needed to validate glyphosate-specific assumptions used in estimating internal dose to more accurately quantify exposure and to harmonise estimates derived from biomonitoring and passive dosimetry.
